# In Vivo Pathogenicity of Methicillin-Susceptible *Staphylococcus aureus* Strains Carrying Panton–Valentine Leukocidin Gene

**DOI:** 10.3390/life12122126

**Published:** 2022-12-16

**Authors:** Funda Yildirim, Mert Sudagidan, Ali Aydin, Ibrahim Akyazi, Gulay Merve Bayrakal, Orhan Yavuz, Aydin Gurel

**Affiliations:** 1 Department of Pathology, Faculty of Veterinary Medicine, Istanbul University-Cerrahpasa, Istanbul 34500, Turkey; 2 Scientific and Technology Application and Research Center, Mehmet Akif Ersoy University, Burdur 15030, Turkey; 3 Department of Food Hygiene and Technology, Faculty of Veterinary Medicine, Istanbul University-Cerrahpasa, Istanbul 34500, Turkey; 4 Department of Physiology, Faculty of Veterinary Medicine, Istanbul University-Cerrahpasa, Istanbul 34500, Turkey

**Keywords:** Panton–Valentine leukocidin, methicillin-susceptible *Staphylococcus aureus*, pneumonia, immunohistochemistry, ELISA, pulsed-field gel electrophoresis

## Abstract

Toxin-producing *Staphylococcus aureus* strains posing a potential risk for public health have long been a topic of scientific research. Effects of Panton–Valentine leukocidin (PVL) on tissue destruction mechanisms and activities of inflammatory cells were presented in animal models of pneumonia and skin infections induced by PVL-producing *S. aureus* strains. This study aimed to demonstrate the in vivo pathogenicity of PVL-producing *S. aureus* strains isolated from some foodstuffs, which can be a potential risk to public health. PVL-positive methicillin-susceptible *S. aureus* (MSSA) strains M1 and YF1B-b isolated from different foodstuffs and a PVL-positive MSSA strain HT480 (positive control) were administered to New Zealand rabbits. Blood samples were harvested three and six hours after the intratracheal inoculation. Lung tissue samples were collected for gross and microscopic exams and immunohistochemical (IHC) demonstration of IL-6, IL8, IL-10, and TNF-α expressions. Serum cytokine levels were also measured by ELISA. The strains isolated from lung tissue samples were confirmed by pulsed-field gel electrophoresis. The development of acute necrotising pneumonia and a significant elevation in IL-6, IL-8, IL-10, and TNF-α expressions demonstrated the significance of foodborne PVL-positive MSSA strains in public health for the first time.

## 1. Introduction

*Staphylococcus aureus* (*S. aureus*) is a significant pathogen for humans and animals, causing a wide range of diseases, such as skin and soft tissue infections, necrotising pneumonia, endocarditis, bacteraemia, and food poisoning [1,2,3,4,5]. Toxins (haemolysins and leukocidins), immune-evasive surface factors (surface protein A), and enzymes that promote tissue invasion, such as hyaluronidase, are the major virulence factors of *S. aureus* [6]. Panton–Valentine leukocidin (PVL) produced by *S. aureus* is a bicomponent exotoxin that creates pores on the membranes of specific immune system cells, particularly polymorphonuclear neutrophils (PMNs) [7,8,9]. The gene (lukSF-PV) encoding PVL comprises two co-transcribed open reading frames (lukS-PV and lukF-PV). It is located on lysogenic bacteriophages, integrated into the chromosome of *S. aureus* [10]. The relation between PVL and some pathologic conditions, such as necrotising pneumonia, soft tissue infections, and neutrophil lysis, has been well known. The association of PVL with the severity of methicillin-resistant *S. aureus* (MRSA) infections was shown in humans and animals [11]. Furthermore, PVL-positive MRSA isolates are significant and frequent causes of necrotising pneumonia [6,12,13,14], and 70–100% of community-acquired MRSA (CA-MRSA) clones have been reported to contain the PVL gene [15].

Bacteriophages undergo high recombination rates, and vertical and horizontal gene transfer allows for genetic change [16]. In previous research, horizontal gene transfer between *S. aureus* strains from different lineages was believed to be rare due to the lineage-specific Type 1 restriction-modification system [17]. However, a later in vivo study revealed that the bacteriophage is frequently transferred by *S. aureus* during co-colonisation. According to this study, recombination has occurred between the same lineage and different bacteriophages [18].

A study of MRSA colonisation in Western Australia revealed that 8% of screening swab sets containing an MRSA were colonised with more than one MRSA strain, and 51.7% were co-colonised with an MSSA [19]. These results indicate ideal opportunities for bacteriophage transmission and recombination among *S. aureus* strains. Therefore, the fact that *S. aureus* strains isolated from foodstuffs or various surfaces carry the PVL toxin gene, located on bacteriophage as mobile genetic material, as well as possible co-colonisation between strains, carries significant risks for public health. Moreover, investigating the PVL-encoding strains and phages among *S. aureus* may help better understand these pathogens’ evolution and may have importance for epidemiology.

MRSA strains are of great clinical significance, and the mortality rate due to virulence factors can be high in hospital-onset cases [6]. Studies have indicated that MRSA strains with various virulence properties, considered more clinically significant, might pose significant risks concerning food hygiene and public health [20,21,22]. MRSA could also be transmitted to humans through food. MRSA strains isolated from hospitals can be foodborne. They may cause intoxication in humans exposed to CA-MRSA strains through foodstuffs or in food industry personnel likely to be infected with the strains carrying high levels of PVL genes [20]. Studies have shown that MRSA colonisation increases the risk of infection through contaminated stuff, such as foodstuffs, production benches, clothes, and mobile phones, in direct contact with the skin, which emerges as fomite contamination, and this colonisation persists for a long time. After transmission, the initial colonisation in the body may not be stable, and different strains can evolve and even be replaced in the same host [6]. In addition to fomite contamination, consuming foods contaminated with bacteria also carries the potential for foodborne gastrointestinal infections. Bacteria colonising at any body site initiate an infection that can develop, spread, and progress to a fatal stage with bacteraemia if treatment is neglected. Therefore, MRSA infections have been researched and have had great importance in public health for a long time.

On the other hand, PVL-positive methicillin-susceptible *S. aureus* (MSSA) has not been commonly detected worldwide, and its role has not been comprehensively examined [23]. However, it has been shown in recent studies that MSSA strains carrying PVL toxin can be at least as potent, infectious pathogens as PVL-positive MRSA strains. Two studies conducted in hospital emergency departments and clinics have shown that skin and soft tissue infections originate from PVL-positive MSSA [24,25]. Varshney et al. [23] reported that the amount of PVL toxin produced in staphylococcal infections is noteworthy. The isolated MSSA has a higher amount of PVL toxin than the MRSA strains that cause soft tissue infections despite the explicit capacity of MRSA strains to produce PVL toxin [23].

Previous studies investigated the presence of PVL genes in 219 *S. aureus* strains isolated from foodstuffs collected in Turkey [26]. MSSA M1 and YF1B-b strains (ST152 MLST type and t355 spa-type), isolated from two different foodstuffs (pasta and phyllo pastry) in the Edirne province (Keşan district), were found to be positive for the PVL gene [22]. This is the first report that has announced the presence of PVL-positive MSSA strain in foodstuffs. The authors also investigated the virulence characteristics of foodborne *S. aureus* strains and other genes with potential virulence effects [27,28]. At this point, the critical question was whether the PVL-positive MSSA-contaminated foodstuffs posed significant risks as MRSA, concerning human and animal health. Therefore, an in vivo study which shows the pathogenic potential of these isolates (M1 and YF1B-b) was planned.

Cytokines and chemokines are essential in mediating, accelerating, and maintaining inflammation. The effect of PVL on cytokine release from neutrophils is controversial. PVL induces the release of some pro-inflammatory mediators such as histamine, leukotriene B4, and IL-8 [29]. When the concentration of PVL toxin is insufficient to create pores in the membrane, PVL toxins trigger the release of IL-8 by activating neutrophils and granulocytes. Furthermore, tumour necrosis factor-alpha (TNF-α) levels were decreased in lung cells of mice by toxin-producing and mutated PVL-positive strains. Yet, those of other crucial inflammatory cytokines remained unchanged [30]. A significant increase was noted in IL-6, IL-8, and TNF-α levels 5 h after the exposure of human neutrophils to purified recombinant PVL (rPVL) toxins, in correlation with the PVL concentration. The study results indicated that rPVL triggered the release of pro-inflammatory cytokines by neutrophils involved in tissue destruction. On the other hand, various concentrations of rPVL were demonstrated to exert no effect on IL-10 release from human neutrophils [31].

In the present study, a pneumonia model was established in rabbits using PVL-positive *S. aureus* strains isolated from foodstuffs to determine in vivo pathogenicity of these strains leading to pneumonia or not both grossly and histopathologically compared to the naïve group and positive control strain. Furthermore, pro-inflammatory and anti-inflammatory cytokines (IL-6, IL-8, IL-10, and TNF-α) were also investigated immunohistochemically and serologically. Finally, the presence of inoculated test strains in lung tissue was determined by PFGE analysis.

## 2. Materials and Methods

### 2.1. Ethical Aspects

The experiments were carried out according to the Turkish guidelines (in completion with NIH guidelines) audited by the Animal Experiments Centre Ethics Committee of the Ministry of Agriculture and Forestry, Republic of Turkey. The ethical approval was obtained from the Local Ethics Committee of Istanbul University with document number 2011/83 (dated 30 June 2011).

### 2.2. Test and Control Strains 

This study used previously isolated and characterised foodborne MSSA strains (M1 and YF1B-b) as test strains [26]. PVL-positive MSSA control strain, HT480, was kindly provided by Prof. Jerome Etienne, Faculté de Médecine Laennec, Equipe Pathogénie des Staphylocoques, France. All strains were stored at −80 °C in 20% (*v*/*v*) glycerol stock solutions.

### 2.3. Preparation of Culture Medium 

The casein–casein–yeast (CCY) medium was prepared to contain 30 g/L yeast extract (Oxoid, Basingstoke, UK), 20 g/L casamino acids (VWR Amresco, Cleveland, OH, USA), 2.48 g/L Na_2_HPO_4_, 0.41 g/L KH_2_PO_4_, 20 mg/L MgSO_4_·7H_2_O, 7.5 mg/L MnSO_4_·H_2_O, 6.4 mg/L citric acids, and 6.4 mg/L FeSO_4_·7H_2_O (Sigma, Darmstadt, Germany) (pH 7.3 ± 0.2 at 25 °C). Then, a 100 mL sodium pyruvate solution was prepared by dissolving 23.2 g of sodium pyruvate (Sigma) in 100 mL deionised water, sterilised by 0.22 µm filtration, and added to 900 mL autoclaved CCY medium at 121 °C for 15 min [32]. *S. aureus* test strains, M1, YF1B-b, and *S. aureus* control strain HT480, were grown on a Tryptone Soy Agar (CM131, Oxoid) at 37 °C by overnight incubation. Bacterial suspensions were prepared in 0.9% (*w*/*v*) NaCl (Sigma) and adjusted to McFarland 0.5 using a densitometer (Den-1B, Biosan, Riga, Latvia). Each bacterial suspension (500 µL) was inoculated to a 50 mL CCY medium and incubated at 37 °C for 24 h by shaking at 120 rpm to promote PVL production. 

### 2.4. Animal Model

A total of 40 10-month-old New Zealand white rabbits of either gender were obtained from the Uludag University Experimental Animal Breeding, Application, and Research Centre, Bursa-Turkey. Food and water were provided ad libitum throughout the study. Animals were divided into five groups, each containing eight rabbits as naïve control (Group I), vehicle control (only CCY medium: Group II), positive control (*S. aureus* HT480 strain: Group III), and two challenge groups (*S. aureus* M1 strain: Group IV and *S. aureus* YF1B-b strain: Group V). All strains were grown in CCY medium (average 5 × 10^8^ CFU/mL), and 1.5 mL from each bacterial culture was applied to the positive control and challenge groups by intratracheal injection to determine pneumonia development in the lungs. Moreover, a 1.5 mL CCY medium was injected into the vehicle control group, and a 96 h incubation period was allowed [33]. After the intratracheal inoculation, the rabbits were monitored, and blood samples were collected on the 3rd and 6th h. Before the cessation of the incubation period, those who died for any reason were instantly necropsied. All surviving animals were euthanised at the end of 96 h. The euthanasia was started with the intramuscular injection of ketamine hydrochloride (20 mg/kg) and xylazine (5 mg/kg) combination for anaesthesia. Then, each rabbit was placed in a supine position, and anaesthesia was maintained via continuous intravenous (i.v.) infusion of ketamine-xylazine (10 mg/kg–2 mg/kg). The euthanasia process was completed by adding sodium pentobarbital (i.v., 150 mg/kg). Lung macroscopic examination was performed during the necropsy, and lung tissue samples were collected aseptically for histopathologic and microbiologic analyses.

### 2.5. Histopathology and Immunohistochemistry

Lung tissues harvested from all sacrificed animals were initially fixed in 10% buffered formalin (103999, Merck, Darmstadt, Germany), routinely processed, embedded in paraffin, sectioned at 5-µm-thickness by a rotary microtome (RM2255, Leica Biosystems, Deer Park, IL, USA), and finally stained with haematoxylin and eosin (H&E) to be evaluated by a light microscope (BX50, Olympus, Tokyo, Japan). Histopathological parameters indicative of tissue injury and inflammation in the lungs, such as oedema, haemorrhage, exudate/fibrin exudate, PMN infiltration, and necrosis, were scored based on a 4-point scale as follows: none (0), mild (1), moderate (2), and severe (3) [33].

Primary antibodies directed against IL-6, IL-8, IL-10, and TNF-α were used for immunohistochemical staining. Lung sections were initially collected on positively charged glass slides, deparaffinised in xylene (108661, Merck), hydrated gradually through graded alcohols (100%, 96%, 80%, and 70% (*v*/*v*), respectively), and finally in distilled water. For antigen retrieval, slides were treated with citrate buffer (pH 6.0) for 20 min in a microwave oven at a high temperature (750 W) and then left to cool. Endogenous peroxidase activity was blocked by the 0.3% hydrogen peroxide in methanol for 10 min at room temperature. Then, the slides were placed in a humid chamber and covered with a blocking solution for 10 min to prevent non-specific binding. Subsequently, slides were incubated with the following primary antibodies for 90 min at room temperature: Polyclonal anti-IL-6 (MBS2006773, 1:200 dilution, Mybiosource, San Diego, CA, USA), monoclonal anti-IL-8 (MBS2025703, 1:200 dilution, Mybiosource), monoclonal anti-IL-10 (MBS2090481, 1/200 dilution, Mybiosource), and monoclonal anti-TNF-α (MBS438099, 1:200 dilution, Mybiosource). After the incubation with the primary antibody, the protocol proceeded with a commercially available detection kit (Expose Mouse and Rabbit Specific HRP/DAB Detection IHC Kit, ab80436, Abcam, Waltham, MA, USA) according to the manufacturer’s instructions. During anti-IL-6 and anti-IL-10 staining, goat anti-guinea pig IgG conjugated HRP (ab6908, Abcam) was used as the secondary antibody. Immunolabelling was visualised by 3,3-diaminobenzidine (DAB) as the chromogen. Finally, the slides were counterstained with Mayer’s haematoxylin. Tween 20 (822184, Merck) added buffered phosphate solution (pH 7.4) was applied as the rinsing solution between each step, starting from antigen retrieval to counterstaining throughout the whole staining protocol, except for the step before the incubation with primary antibodies, which were replaced by the rinsing solution for the negative controls.

The scoring system of immunohistochemical analysis was based on the proportion of the immunolabelled cells and the staining intensity. According to the proportion of immunollabeled cells: 0%, none (0); up to 30%, mild (1); 30–60%, moderate (2); over 60%, severe (3). As for the positive reaction intensity: no reaction (0); slight (1); moderate (2); severe (3). The final score was determined by multiplying these two scores. The numerical results were expressed as negative (0), mild (1–3), moderate (4–6), and strongly positive (7–9) [34]. The final scores were statistically assessed as 0 = negative, 1 = mild, 2 = moderate, and 3 = strong.

### 2.6. Cytokine Levels

Blood samples were collected 3 and 6 h after intratracheal inoculation of the strains. Sera were obtained and then stored at −80 °C. ELISA kits were used to measure IL-6 (CSB-E 06903Rb), IL-8 (CSB-E 06905Rb), IL-10 (CSB-E 06897Rb), and TNF-α (CSB-E 06998Rb, Cusabio, Houston, TX, USA) levels. The plates were read by an ELISA reader (RT-6000 Rayto, Shenzhen, China) at 450 nm.

### 2.7. Isolation of Inoculated Strains

The lung tissue samples of the rabbits were collected into sterile tubes to isolate bacterial strains. A lung tissue portion of 3 × 3 mm was taken aseptically and added into 10 mL Brain Heart Infusion Broth (BHI, CM1135, Oxoid). The bacteria were grown at 37 °C by shaking at 120 rpm for 24 h. Then, the grown bacteria were inoculated onto Mannitol Salt Agar (MSA, CM085, Oxoid) and incubated at 37 °C for 48 h to isolate *S. aureus* strains. Presumptive *S. aureus* strains were selected from MSA and inoculated into Tryptone Soy Broth (TSB, CM129, Oxoid). Then, 20% (*v*/*v*) glycerol stock solutions were prepared and stored at −80 °C for further analysis. DNA extraction from presumptive *S. aureus* strains was carried out as described by Sudagidan et al. [35]. *S. aureus* strains were identified by PCR using specific primers for *nuc* [36], *coa* [37], and *spa* genes [38]. The presence of the PVL gene in the isolated strains was searched by PCR using lukSF-PV-specific primers [39].

### 2.8. Pulsed-Field Gel Electrophoresis

The isolated *S. aureus* strains from lung tissues and inoculated test and control *S. aureus* strains were compared using PFGE analysis. For this purpose, agarose plugs were prepared as previously described by Durmaz et al. [40]. Bacterial DNA in the plug was digested by 30 U Smal (Thermo, Waltham, MA, USA) at 30 °C for 16 h, and the plugs were loaded into 150 mL 1% (*w*/*v*) PFGE grade agarose gel (Bio-Rad, Hercules, CA, USA). PFGE gel was run in 0.5× TBE buffer at 14 °C for 22 h, 5–40 s pulse time, 120°, and 6 V/cm current using CHEF-DR II PFGE system (Bio-Rad). After electrophoresis, PFGE gel was stained by 2.5× EZ-Vision DNA dye (VWR Amresco), and the bands were visualised in the Gel Doc™ EZ gel documentation system (Bio-Rad). The obtained band patterns were analysed by BioNumerics Version 7.6 (AppliedMaths, Sint-Martens-Latem, Belgium).

### 2.9. Statistical Analysis

The nonparametric tests, Kruskal–Wallis, and Mann–Whitney U, were performed for statistical evaluation of histopathological and immunohistochemical scores. ELISA measurements performed at 3 and 6 h to determine cytokine concentrations were analysed using one-way ANOVA. The data regarding the correlation between cytokine expression and the first and second measurement times were compared among and within each group individually for each cytokine to assess group averages and the standard error. Furthermore, the significance of the group and measurement times were assessed by the repeated measurement analysis of variance. The significance level was determined as *p* < 0.05. All statistical analyses were performed using the SPSS software version 13.0 (IBM, Armonk, NY, USA).

## 3. Results

### 3.1. Animal Response

**Figure 1:**
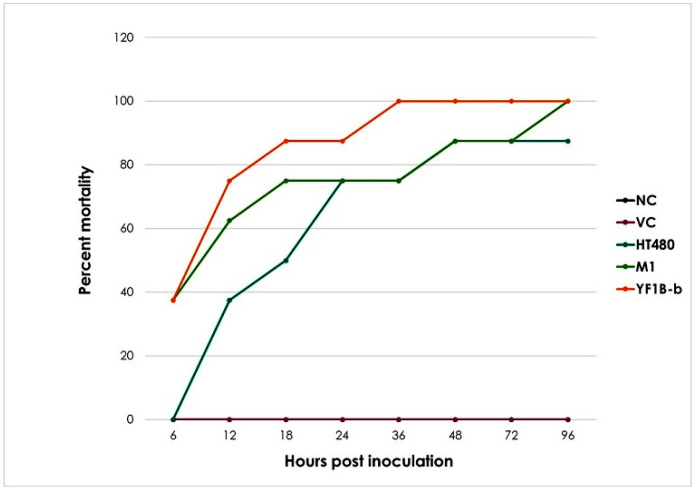
Time-dependent mortality rate change. NC: naïve control, VC: vehicle control, HT480: PVL-positive MSSA control strain, M1: foodborne MSSA strain isolated from pasta, and YF1B-b: foodborne MSSA strain isolated from phyllo pastry.

The rabbits in Groups I and II survived up to the 96th h with no clinical symptoms, and they were sacrificed once the inoculation period had ended. In Group III, to which the positive control strain was applied, four of eight individuals developed a high fever and wheezing in the first 13 h of inoculation. Two of them manifested the relevant clinical signs at 18–21 h. An animal died at the 40th h of inoculation, and only a single rabbit from this group survived up to the 96th h. In Group IV, five of eight individuals had a high fever and wheezing and died within the first 11 h of inoculation, while two died at the 18th and 20th h. The rest of the group died at the 83rd h of inoculum. Group V, given YF1B-b, six of eight individuals who developed a high fever and wheezing after inoculation died in the first ten hours, while two died after 14.5 and 35.5 h. The mortality chart of the animals is shown in Figure 1.

### 3.2. Gross Pathological Findings

**Figure 2:**
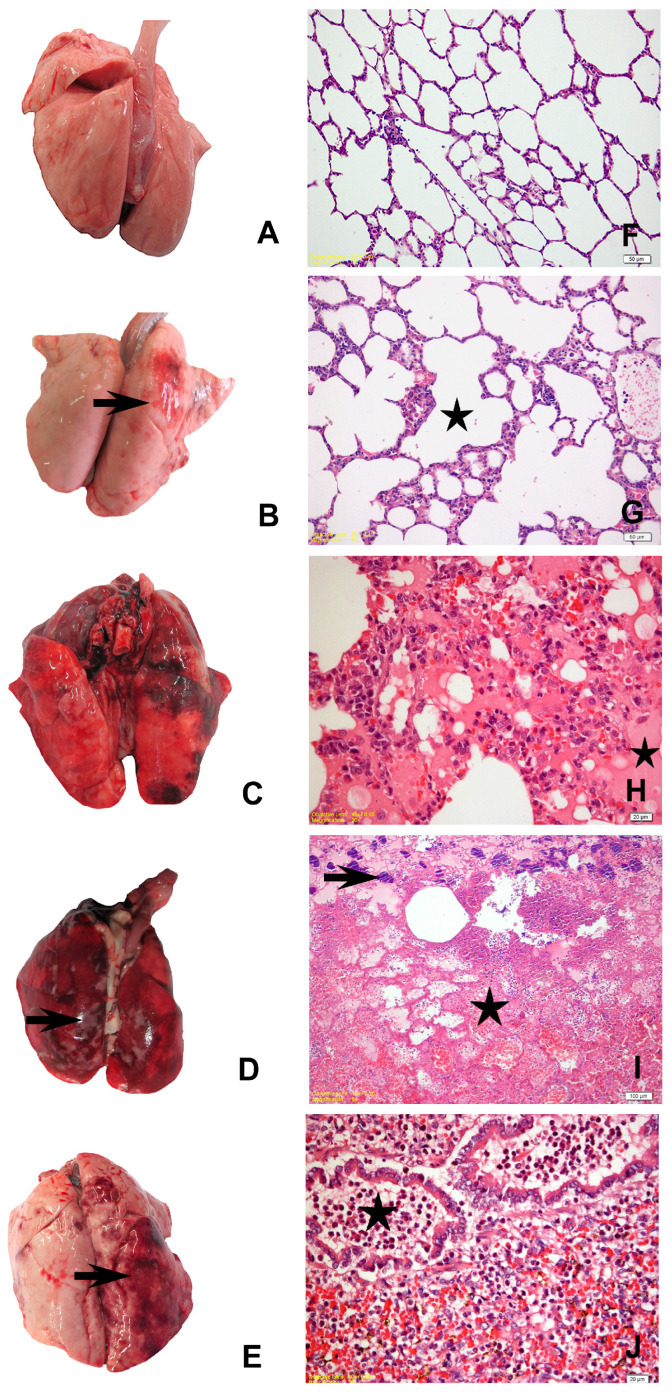
(**A**) Mild pulmonary oedema and emphysema, naïve control group; (**B**) mild pulmonary oedema and congestion (arrow), vehicle control group; (**C**) diffuse hyperaemia and foci of haemorrhage in the right lung lobes, positive control group; (**D**) widespread hepatisation, haemorrhage, and pale foci of necrosis (arrow) in the entire lung tissue, M1 trial group; (**E**) diffuse oedema, emphysema, focal haemorrhage, and hepatisation in the right lung lobes (arrow), YF1B-b trial group; (**F**) healthy lung parenchyma of the naïve control group; (**G**) mild alveolar emphysema (star), vehicle control group; (**H**) hyperaemia, oedema (star), and alveolar macrophage proliferation, positive control group; (**I**) areas of massive necrosis (star), erythrocytes in the alveolar lumina, fibrinous exudate, karyorrhectic PMNs, and bacterial colonies (arrow), M1 Group; (**J**) dense PMN infiltration in the bronchioles (star) and alveolar lumina, hyperaemia, and alveolar macrophage proliferation, YF1B-b group. All sections were stained withH&E; (**F**,**G**) bar = 50 µm; (**H**,**J**) bars = 20 µm; (**I**) bar = 100 µm.

No macroscopic changes were observed in the lungs of the rabbits in Group I (naïve control) and II (vehicle control), except for a few animals from the vehicle control group that had mild oedema and emphysema (Figure 2A,B). In the positive control group (Group III), animals that died in the first 13 h had diffuse emphysema, oedema, hyperaemia, and focal areas of petechial haemorrhages (Figure 2C). Widespread hepatisation and pale foci of necrosis were observed on the lung surface in rabbits that died between 18–40 h. No significant change was observed macroscopically in the euthanised animal at the 96th h. Nasal haemorrhagic discharge was observed in two animals in Group IV that received the M1 strain. Blood had a darker colour and appeared viscous in all animals. Grossly significant emphysema, oedema, mild bleeding, and areas of hepatisation were detected in the lungs. Widespread hepatisation and 2–3 mm foci of necrosis (Figure 2D) were noted in the lungs of the individuals who died at the 83rd h. Like other challenge groups, the blood was darker and had a thicker consistency in all individuals of Group V (YF1B-b strain inoculated group). Macroscopically distinct emphysema, oedema, some mild bleeding, and hepatisation areas were detected in the lungs of all animals (Figure 2E).

### 3.3. Histopathological Findings

There was no microscopic change in the naïve control group (Figure 2F). Except for mild congestion and oedema in some individuals in the vehicle control group, no significant difference was observed in the control groups (Figure 2G). Similar histopathological changes were detected in the individuals in all trial groups. Widespread oedema, congestion, bleeding, mild leukocyte infiltration, and alveolar macrophage proliferation were observed in the lungs of those who died within the first 7–8 h after the bacteria were administered in all trial strains, including the positive control group. Focal areas of emphysema, oedema, congestion, diffuse bleeding, massive necrosis, severe neutrophil leukocyte infiltration, fibrinous exudate, and necrotic pneumonia were detected in the lungs of individuals who survived longer (Figure 2H,J). There were also bacterial colonies within the scattered necrosis areas in a few animals’ lung tissue sections (Figure 2I).

The comparative evaluations based on the scoring of histopathological parameters revealed significant differences between the control and challenge groups. However, in paired group comparisons due to oedema and PMN infiltration in some vehicle group individuals, no significant difference was detected between the YF1B-b and vehicle control groups regarding the relevant two histopathologic parameters. The statistical analysis results of the histopathological evaluations were summarised in Table 1 and Table 2.

### 3.4. Immunohistochemical Findings

**Figure 3:**
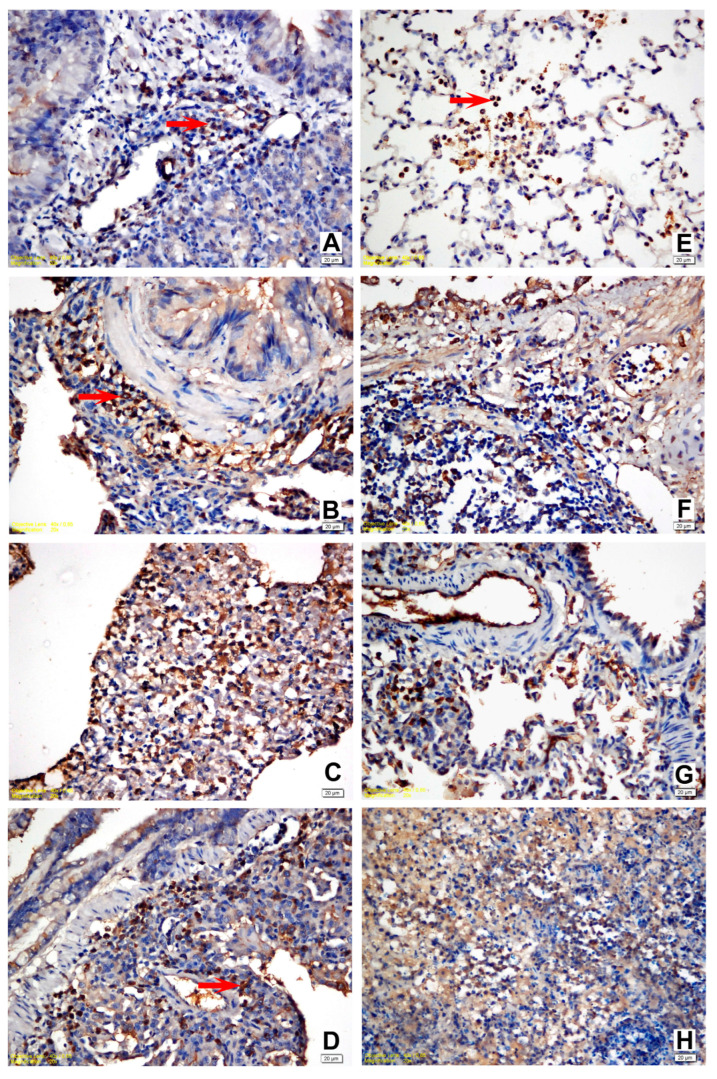
(**A**) Slight positivity for IL-6 in the lymphoid and mononuclear cells within the peribronchial area, vehicle control group; (**B**) strong positivity for IL-6 in all mononuclear and lymphoid cells within the peribronchial area, positive control group; (**C**) strong positivity for IL-6 in all mononuclear, fibroblastic, and endothelial cells within the peribronchial area, M1 trial group; (**D**) strong positivity for IL-6 in all mononuclear and lymphoid cells within the peribronchial area, YF1B-b trial group; (**E**) moderate positivity for TNF-α in the activated alveolar macrophages and PMNs, vehicle control group; (**F**) strong positivity for TNF-α in the lymphoid and mononuclear cells within the peribronchial area, positive control group; (**G**) strong positivity for TNF-α in all mononuclear, fibroblastic, and endothelial cells within the peribronchial area, M1 trial group; (**H**) moderate positivity for TNF-α within the area of PMN infiltration. The majority of PMNs are karyorrhectic, YF1B-b group. Mayer Haematoxylin was used for background staining. Arrows show positive staining cells. All bars = 20 µm.

**Figure 4:**
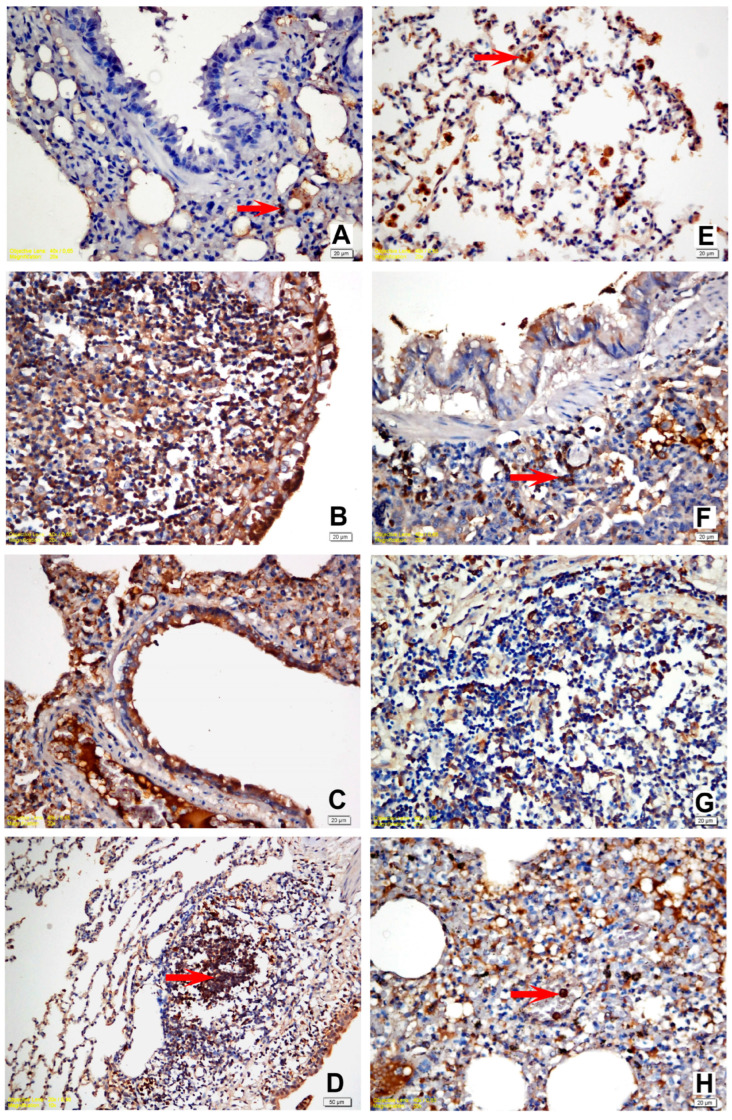
(**A**) Slight positivity for IL-8 in a few mononuclear cells, vehicle control group; (**B**) strong positivity for IL-8 in all mononuclear and lymphoid cells within the peribronchial area and the bronchial epithelial cells, positive control group; (**C**) strong positivity for IL-8 in all mononuclear, fibroblastic, endothelial cells, and bronchial epithelial cells, M1 trial group; (**D**) strong positivity for IL-8 in all mononuclear and lymphoid cells within the peribronchial area. Alveolar and bronchial epithelial cells were also intensely stained, YF1B-b trial group; (**E**) moderate positivity for IL-10 in the activated alveolar macrophages and PMNs, vehicle control group; (**F**) strong positivity for IL-10 in the peribronchial lymphoid and mononuclear cells, positive control group; (**G**) strong positivity for IL-10 in the peribronchial lymphoid and mononuclear cells, M1 trial group; (**H**) moderate positivity for IL-10 in PMNs and alveolar macrophages, YF1B-b group. Mayer Haematoxylin was used for background staining. Arrows show positive staining cells. (**D**) bar = 50 µm; in all other figures: bar = 20 µm.

The IHC staining for IL-6, IL-8, IL-10, and TNF-α expression in the lung tissue revealed a slight intensity in a few animals in Group I while moderate in Group II. A moderate to strong positive reaction was observed with all cytokine markers in all challenge groups (Figure 3 and Figure 4). Statistically significant differences were noted between the naïve group (Group I) and all other experimental groups. A statistically significant difference (*p* = 0.020) was detected solely in terms of TNF-α staining between Group II and Group III since TNF-α expression revealed an intense immune reaction in the positive control (Group III). Apart from the relevant significant difference, no other differences were noted between the vehicle group (Group II) and the challenge groups regarding immunohistochemical expression scores. The results of the statistical analysis of immunohistochemical staining are shown in Table 3 and Table 4.

### 3.5. ELISA Results

**Figure 5:**
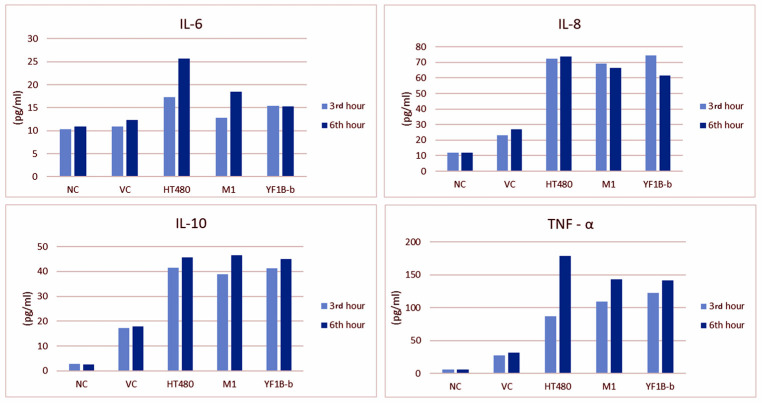
ELISA results of IL-6, IL-8, IL-10, and TNF-α levels (pg/mL) in the blood samples collected at the 3rd and 6th h of inoculation.

Serum IL-6, IL-8, IL-10, and TNF-α levels in all groups after challenge (at the 3rd and 6th h of inoculation) are presented in Figure 5. The 6th h measurements regarding IL-6, IL-10, and TNF-α levels were higher in all groups except for Group I. IL-6 and TNF-α levels were noted to have increased significantly in the positive control (Group III) at the 6th h. Unlike other cytokine levels, IL-8 measurements revealed a decrease in the second measurement time point (at the 6th h) in all groups except for Group III.

The comparisons between the first and second measurement times for all parameters in all experimental groups revealed an increase merely for TNF-α level at the second measurement time in Group III (*p* = 0.011) and Group IV (*p* = 0.010), which was found to be statistically significant. While the measurement time for IL-6 and TNF-α levels emerged as an essential variable by the repeated ANOVA tests, the group variable was statistically significant for all cytokines. The results of the statistical analyses regarding ELISA measurements are presented in Table 5.

### 3.6. Isolation of Inoculated Strains and PFGE Analysis

**Figure 6:**
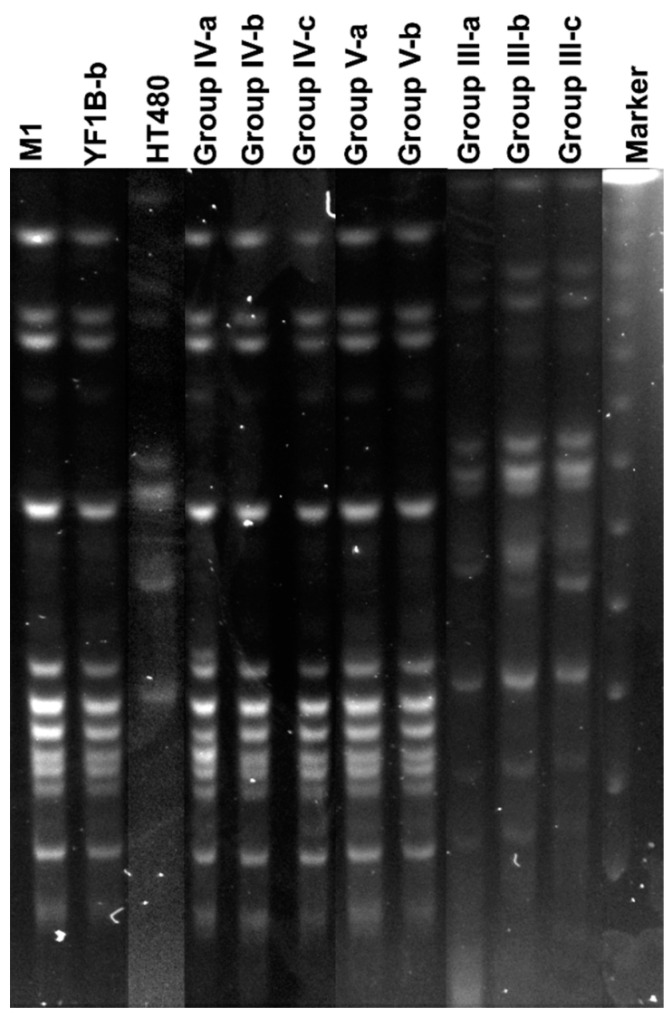
PFGE band patterns of inoculated *S. aureus* strains (M1, YF1B-b, and HT480) and the isolated *S. aureus* strains (n:8) from rabbit lung tissues (Group IV-a, b, c, Group V-a, b and Group III-a, b, c). The marker was a lambda ladder for PFGE (Bio-Rad).

The bacteria in rabbit lungs (n:12) were pre-enriched in BHI, and *S. aureus* strains were selected that were grown on MSA. The colonies on MSA were purified, and single colonies were inoculated to TSB for DNA extraction. The isolated bacteria were identified by PCR using primers for a *nuc*, *coa*, *spa*, and PVL genes. *S. aureus* could not be isolated in the vehicle control containing only CCY medium. PCR results of isolates showed that 8/9 isolates contained *nuc*, *coa*, *spa*, and PVL genes. Only one isolate from YF1B-b inoculated rabbit lung was not *S. aureus*, and it was PVL negative. In PFGE analysis, the test and control strains (*S. aureus* M1, YF1B-b, and HT480) inoculated into rabbits were compared with isolated PVL-positive *S. aureus* strains. The obtained band patterns were analysed, and the isolated strains from lung tissues proved to be inoculated strains. In Figure 6, the inoculated and isolated strains from the lungs showed the same indistinguishable PFGE band patterns.

## 4. Discussion

*S. aureus* produces six pore-forming toxins, such as α-haemolysin (Hla), β-haemolysin, γ-haemolysin (Hlg), δ-haemolysin, Luk, and PVL [41]. The PVL toxin is encoded by the PVL genes and integrated into the chromosome of MSSA or MRSA [42]. The screening for PVL genes in a collection of clinical *S. aureus* isolates revealed that PVL was associated with deep skin infections and severe forms of primary community-acquired pneumonia with haemorrhagic and necrotic features [39]. Gillet et al. [43] stated that PVL was isolated from approximately 5% of cases caused by *S. aureus*. Apart from PVL-negative *S. aureus* strains, the disease caused by PVL-positive *S. aureus* was defined as a separate entity for the first time in a previous study, and the disease was histopathologically referred to as “*S. aureus* necrotising pneumonia” due to the necrotic appearance in the lungs [43].

Genetically diverse PVL-positive MSSA and MRSA clones have been detected worldwide. PVL-positive MSSA reveals the same spectrum of disease as PVL-MRSA and has similar epidemiological characteristics. They are associated with skin and soft tissue infections but can also cause life-threatening conditions such as necrotising pneumonia and necrotising fasciitis [44]. Community-associated MRSA (CA-MRSA) isolates are an emerging concern. The evolution of CA-MRSA in persons with healthcare-associated risk factors was initially reported in the early 1980s among hospital employees in the United States [45]. MRSA still poses a significant clinical threat, with persistently high morbidity and mortality [6], even though its incidence has recently declined in some regions of the world. Similarly, PVL-positive MSSA isolates are significant due to their potential risks. A recent study has shown that PVL-positive MSSA led to an outbreak in a neonatal unit in northwest London, UK. In the study, several PVL-positive MSSA were cited in maternity and neonatal units in England and France [44].

Another noteworthy point is that *S. aureus* is considered one of the most common pathogens responsible for food poisoning outbreaks. Methicillin-resistant *S. aureus* (MRSA) has become a leading cause of nosocomial infections and one of the most prevalent pathogens worldwide, with a significant economic impact on healthcare systems.

Recently, MRSA has become a zoonotic issue since a particular MRSA strain of sequence type 398 (ST398) has been frequently detected in pigs and pig handlers. Several reports have demonstrated an elevated prevalence of MRSA among pig handlers, which suggests a presumable regional emergence of CA-MRSA [46]. Thus, the infections caused by PVL-positive *S. aureus* strains are still a potential menace to public health.

MRSA colonisation increases the risk for infection, and the strains isolated from at least 50–80% of the infected patients referred to the hospital correspond with the strains colonised in different environments [47]. Exposure to contaminated items, including scrubs, ties, pens, and cell phones through direct skin contact, plays a significant role in MRSA transmission. “Bacterial colonisation” is a process that sustains for extended periods. MRSA tends to persist within the home environment, which, as a result, hampers its eradication [48]. Likewise, MSSA strains can colonise several surfaces, leading to an onset of an infection. Desai et al. [49] showed that CA-MRSA, hospital acquired-MRSA, and CA-MSSA strains were transmitted through nine different fomites, indicating that CA-MRSA strains survived longer on the surfaces of fomites than CA-MSSA strains.

Foods frequently contaminated with staphylococcal species mainly comprise poultry/egg products, meat products, milk/dairy products, and bakery products [50]. Sudagidan and Aydin [26,36] screened several foodstuffs for potential PVL-positive *S. aureus* strains and isolated the first worldwide foodborne PVL-positive MSSA strains. In later studies, the authors asserted the prevalence of the PVL gene, its virulence effects, and virulence properties [27,28]. However, whether these foodborne PVL-positive strains, which are negative for toxin genes (*sea*, *seb*, *sec*, *sed*, *see*, *eta*, *etb*, and *tst* genes) [26], can cause a severe infection such as potential necrotising pneumonia in humans and animals, is a crucial issue concerning public health and requires a thorough investigation by in vivo studies.

In this study, a pneumonia model was established in rabbits, recognised as the most susceptible animal species to PVL toxin [33], by provoking the toxin production from the strains grown in CCY medium, the most suitable medium to stimulate PVL production by bacteria [51]. Interestingly, mortality increased by over 60% within the first 24 h in both challenge groups and the positive control in our study. In a study conducted by Montgomery and Daum [52], a pneumonia model was created in rats with a 50% lethal dose (5 × 10^8^ CFU) of SA300 CA-MRSA LAC strain (wild type) and an isogenic lukSF-PV deletion mutant LAC Δ*pvl* strain to examine the host response. Early deaths were observed 5–6 h after inoculation in both groups, and after 24 h of incubation, mortality rates were 62% and 72% in the LAC and LAC Δ*pvl* groups, respectively. In another study, in mice, necrotic pneumonia was induced by PVL-positive and PVL-negative *S. aureus* strains, and the mortality rate varied between 35% and 80% within the first 24 h of the PVL-positive strain inoculation [53]. According to Diep et al. [33], the mean survival time was 3.9 days in a rabbit bacteraemia model challenged by a mixture of wild-type and isogenic Δ*pvl* mutant CA-MRSA strains in an approximate 1:1 ratio. In a subsequent study conducted by Diep et al. [33] in the outbred rabbits, no mortality was noted with 1 × 10^8^ and 5 × 10^8^ CFU of SF8300, whereas treatment with 1 × 10^9^, 5 × 10^9^, and 10 × 10^9^ CFU caused 40–100% mortality.

The existence of conflicting reports regarding species susceptibility is a critical issue that requires elucidation concerning the pathogenicity of PVL-positive *S. aureus* infections. It was reported in the studies investigating disease models sensitive to PVL toxins (human cells and rabbit models) that PVL plays a pathogenic role in the course of the diseases, rendering severe necrosis [33,51]. However, it was shown in experimental studies using murine models that mice are resistant to the pathogenic effects of PVL [54,55]. Löffler et al. [56] investigated the cytotoxic activity of PVL against the neutrophils of humans, rabbits, and Java monkeys. The authors indicated that PVL induced rapid activation and cell death in human and rabbit neutrophils, whereas murine or simian cells remained unaffected. Compared with murine models that are relatively insensitive to the cytotoxic effects of PVL [52,55,56,57], the high mortality noted in the present study proved the rabbit’s susceptibility to PVL toxin. In a recent study, Stulik et al. [58] stated that *S. aureus* pneumonia in humans is undoubtedly not as severe as in rabbits, highlighting that intratracheally applied bacteria are systemically disseminated in lethal rabbit models, representing an exaggerated version of the *S. aureus* pneumonia’s main features. The authors also indicated that most humans express *S. aureus*-specific antibodies, particularly against surface antigens, which are not present in naïve animals [58].

In a rat pneumonia model established by Montgomery et al. [59] for CA-MRSA infection, the lungs of most animals infected with USA400 strains appeared dark pink and mottled, with no evidence of necrosis. Furthermore, the lung tissues of animals infected with USA300 strains, and several animals infected with USA400 strains at the high inoculum, which all survived to 42 h, exhibited necrotic areas distinctive on the haemorrhagic lungs. It was also noted that the lungs were macroscopically congested in all animals. Montgomery and Daum [52] carried out a time-course (based on a monitoring design of 3, 6, 9, and 12 h after infection) experiment on rats, challenged with USA 300 LAC (wild type) and LAC Δ*pvl* (deleted lukS-PV gene) of CA-MRSA strains. The authors showed that severe necrotising pneumonia was present in 57% and 55% of the animals infected with LAC and LAC Δ*pvl*, respectively, indicating that the lungs’ group-wise histopathologic appearances were indistinguishable. The severity of histopathological changes increased 9 h after infection, resulting in severe pneumonia with marked pulmonary oedema, necrosis, and multifocal bacterial aggregates in 60% (6/10) of the inoculated animals at the 9th and 12th h [52]. Labanderia-Rey et al. [53] noted enhanced neutrophil recruitment, a significant inflammatory response in the lung parenchyma, bronchial epithelial damage, tissue necrosis, and pulmonary haemorrhage in mice infected with PVL-positive strains 48 h after inoculation. Diep et al. [33] induced a rabbit pneumonia model by inoculating the wild-type and the Δ*pvl* strain of USA300 MRSA. They found that the wild-type strain caused more extensive necrosis with disrupted pulmonary architecture, haemorrhagic infiltration, exudate/fibrin deposition, alveolar and interstitial oedema, PMN infiltration, and tissue destruction than the effects of the Δ*pvl* strain 48 h after inoculation. In the presented study, remarkable histopathologic alterations were noted in all challenge groups. Histologic evaluation revealed necrotic pneumonia, characterised by necrosis, diffuse bleeding, fibrinous oedema, and severe PMN infiltration in the lung parenchyma. Even though there was no statistical difference among the challenge groups, the positive control strain’s pathogenicity was slightly higher than the test strains based on the paired group comparisons regarding the mean values of histopathological scores.

Additionally, the macroscopic and microscopic findings in the presented rabbit pneumonia model induced by two foodborne *S. aureus* strains were consistent with those in the previous experimental models. Moreover, a few animals from each bacterial strain group revealed intense karyorrhexis of PMNs in the areas of extensive necrosis in which bacterial colonisation was also noted. In a previous study, rPVL was suggested to have induced apoptosis at 10 nmol/L, whereas a 100 nmol/L concentration exerted a necrotic effect on neutrophils [60]. Laventie et al. [61] developed antibodies specific for the LukS-PV and lukF-PV regions of the bacterial strains. In vitro and in vivo experiments showed that these antibodies inhibited the PVL toxin’s adhesion to the immune system cells and prevented pore formation on the cellular membranes. Furthermore, the administered antibodies were shown in vivo to have restrained the PVL-induced inflammation and tissue damage in the rabbits.

Widespread inflammatory response and influx of inflammatory cells to the infection site are observed during necrotic diseases. The phagocytic leukocytes, particularly PMNs, targeted by the PVL toxin, generate the principal defence mechanism of the immune system involved in *S. aureus* infections [56]. Virtually, PVL rapidly kills the recruited immune cells. Active components such as proteases stored within the neutrophils are unrestrainedly released to the surrounding lung tissue, resulting in massive tissue destruction indicative of necrotising pneumonia [56]. Our results confirmed that necrotising pneumonia was mainly due to toxin-mediated alterations in the rabbit lungs.

The effect of PVL on cytokine release from neutrophils has been controversial in previous studies [9,31,52,60,62]. Although the release of cytokines from the immune cells was associated with the cell lysis induced by the PVL toxin, Ma et al. [31] suggested that rPVL significantly increases NF-κB expression in neutrophils, and NF-κB protein is a prerequisite for the release of pro-inflammatory cytokines from several cells (i.e., neutrophils, T cells, and macrophages). Tissue destruction in the lungs occurs solely due to the release of pro-inflammatory cytokines caused by PVL-induced cell lysis and regardless of bacterial cell proliferation [31].

The expression of several cytokines, chemokines, and receptor genes involved in inflammation induced by the intratracheal administration of the sublethal levels of the PVL-positive USA300 CA-MRSA strain was shown to have rapidly increased in rats by quantitative Real-Time PCR analysis. The findings demonstrated that the elevation in gene expression, followed by inflammation and an increased number of bacteria in the lungs, resulted in necrotic pneumonia and substantial tissue destruction [52].

Pro-inflammatory and anti-inflammatory mediators regulate pulmonary inflammation, and PVL toxins induce the release of specific pro-inflammatory agents such as histamine, leukotriene B4, and IL-8 by activating neutrophils and other granulocytes. It has been reported that PVL can trigger an increased (over 16 h) release of the pro-inflammatory cytokine IL-8 from human leukocytes [33,63]. In another study, it was stated that while PVL increased the release of the anti-inflammatory cytokine IL-10, the level of the pro-inflammatory cytokine, TNF-α, released from neutrophil leukocytes was slightly decreased [60]. In contrast, Montgomery and Daum [52] suggested that PVL did not cause a significant change in the transcription of inflammatory genes in the lung in a rat pneumonia model.

In the present study, statistically significant differences were serologically and immunohistochemically determined between the challenge and control groups regarding IL-6, IL-8, TNF-α, and IL-10 expressions. All cytokines were increased in all challenge groups, and those in the vehicle control group were higher than the naïve control group.

IL-6 contributes to the initiation and prolongation of inflammation [64]. Although IL-6, as a pro-inflammatory cytokine, is secreted from several types of cells in the inflammatory region, it has exhibited anti-inflammatory and protective effects by suppressing TNF expression, especially in septic lung injury [33,62]. It was determined in the presented study that the IL-6 level was significantly increased both in the lung tissue and the sera, demonstrated immunohistochemically and by the ELISA tests, respectively. Practically, in all groups, serum IL-6 levels were exceptionally high in the 6th h measurements. However, no statistically significant increase was detected either by group-wise comparisons among the trial groups or measurement time-based comparisons within each group.

IL-8, an effective neutrophil-attracting/activating cytokine, is essential in producing anti-IL-8 autoantibodies in acute lung injury. It has been stated that the expression of IL-8, the most significant cytokine in acute lung injury, increases with the destruction of both pulmonary parenchymal cells and the immune cells that infiltrated the lesion area [33,52]. In the present study, IL-8 expression was increased in all experimental groups, including the vehicle control group, at the first measurement time, and yet, even if not statistically significant, inclined to have decreased at the second measurement time. Conversely, IL-8 levels in the HT480 strain group, which revealed the highest values in the 3rd h measurements, remained unchanged and slightly increased in the second (6th h) measurements. However, there was no significant difference among trial groups regarding IHC staining of IL-8 expression.

Diep et al. [33] induced a pneumonia model in rabbits by the wt PVL-expressing SF8300 strain and measured both lung and serum IL-8 levels while monitoring time-dependent elevation of the relevant cytokine in the lung tissue, and a decrease was determined in the sera after 6 h. Likewise, the 6th h IL-8 levels were decreased in the test strain groups in the present study. The expression of IL-8 induced by PVL in the lung tissue was consistent with the previous studies.

TNF-α is the most widely investigated pleiotropic cytokine of the TNF superfamily. In pathophysiological conditions, the high levels of TNF-α production are responsible for the inflammatory responses considered hallmarks of many diseases [30,62]. In this study, TNF-α was relatively higher in all groups at the second measurement time than at first. However, a statistically significant difference was noted in the HT480 and M1 groups based solely on measurement timewise comparisons within each group. The elevated TNF-α levels in all isolate groups, with the HT480 group revealing the highest value, and the significantly higher scores in terms of IHC staining have indicated the crucial role of TNF-α in PVL-induced lung injury.

IL-10, an anti-inflammatory cytokine, can inhibit the synthesis of pro-inflammatory cytokines, expressed as IFN-γ, IL-2, IL-3, TNF-α, and granulocyte-macrophage colony-stimulating factor by macrophages and helper T cells [52,62]. It was reported that PVL significantly increased the production of IL-10 and slightly decreased the expression of TNF-α by neutrophils [57]. In a study investigating the effects of recombinant PVL on neutrophils, an increase was noted in the PVL gene expression as in IL-6, IL-8, and TNF-α production; however, IL-10 expression remained unchanged. The authors also confirmed that rPVL activates neutrophils to release pro-inflammatory cytokines [31]. PVL has recently been demonstrated to exert a direct cytolytic effect. While it upregulates the expression of pro-inflammatory cytokines, it has no impact on the expression of anti-inflammatory cytokines such as IL-4 and IL-10 [9]. In the presented study, ELISA measurements and immunohistochemical staining scores of IL-10 were significantly higher in the positive control and the two test strain groups than the naïve control group, and the difference between the M1 group and naïve group regarding the IHC staining scores of IL-10 was statistically significant (*p* < 0.001).

In the study, the increased levels of all investigated cytokines (IL-6, IL-8, IL-10, and TNF-α) in the vehicle control group (although relatively lower than the trial groups) were associated with stress exposure, anaesthesia, and intratracheal inoculation-associated trauma. On the other hand, the pro-inflammatory cytokine (IL-6, IL8, and TNF-α) levels revealed the highest values in the positive control group, whereas IL-10 levels in the M1 strain group were higher than all other groups, in terms of both IHC staining scores and the ELISA measurements. Based on the mortality rates, histopathological scores, and cytokine levels data, it might be deduced that the HT480 strain is more potent in pathogenicity than M1 and YF1B-b test strains.

PFGE is a powerful tool to investigate clonal relationships of bacterial strains from the same species. Centres for Disease Control and Prevention (CDC) suggest this gold standard fingerprinting method to identify bacterial clones responsible, especially during outbreaks. In our study, intratracheal inoculated *S. aureus* strains were confirmed with the isolated *S. aureus* strains from lung tissues in rabbits with indistinguishable band patterns obtained in PFGE analysis, which showed that all pneumonia cases in rabbits were caused by inoculated foodborne and control *S. aureus* strains.

## 5. Conclusions

PVL-positive *S. aureus* strains isolated from foodstuffs led to acute lung injury and necrotic pneumonia in the present study. To our knowledge, this is the first experimental pneumonia model induced by the foodborne PVL-positive *S. aureus* strains. We deduced that *S. aureus* strains carrying the PVL gene obtained from foodstuffs showed high pathogenicity in this experimental model, which is crucial for public health due to their potency for natural infection.

Despite the conflicting results of either the disease model (in which the disease models were established in different species) or in vitro experimental studies regarding the role of PVL, the severe necrotising pneumonia developed in the rabbit model induced in the presented research supports the suggestions of species-wise susceptibility to PVL.

Neutrophil leukocytes were shown to play a critical role in lung inflammation and injury induced by *S. aureus* strains carrying PVL of food origin. The study’s IHC and ELISA results supported that neutrophils exacerbate and maintain the acute inflammatory response by releasing pro-inflammatory cytokines. Furthermore, the simultaneous and extensive release of pro-inflammatory and anti-inflammatory cytokines in the PVL-mediated necrotic pneumonia model is another outcome of the present study.

## Figures and Tables

**Table 1:** The effect of group on the scores of histopathological parameters (median (min–max)) in lung sections.

Parameters	Group-I	Group-II	Group-III	Group-IV	Group-V	*p* Value
Oedema	0 (0–0)	0 (0–2)	2 (0–3)	2 (0–3)	1 (0–3)	0.007
Haemorrhage	0 (0–0)	0 (0–0)	2 (0–3)	1.5 (0–3)	1 (0–3)	<0.001
Exudate/Fibrin	0 (0–0)	0 (0–0)	1 (0–2)	1 (0–3)	0.5 (0–2)	0.003
PMN infiltration	0 (0–0)	0 (0–1)	2 (0–3)	2 (0–3)	1 (0–3)	<0.001
Necrosis	0 (0–0)	0 (0–0)	0.5 (0–3)	1 (0–3)	0.5 (0–3)	0.016

**Table 2:** *p* values of the histopathological parameters assessed by the Mann–Whitney U analysis based on paired group comparisons.

Parameters	Group I/Group III (NC/HT480)	Group I/Group IV (NC/M1)	Group I/Group V (NC/YF1B-b)	Group II/Group III (VC/HT480)	Group II/Group IV (VC/M1)	Group II/Group V (VC/YF1B-b)
Oedema	0.004	0.004	0.004	0.071	0.043	0.183 (NS)
Haemorrhage	0.001	0.004	0.003	0.001	0.004	0.003
Exudate/Fibrin	0.010	0.003	0.027	0.010	0.003	0.027
PMN infiltration	0.001	0.001	0.003	0.006	0.006	0.085 (NS)
Necrosis	0.027	0.010	0.027	0.027	0.010	0.027

**Table 3:** Effect of the group on IHC staining scores (median (min–max)) in the lung sections.

Cytokine	Group-I	Group-II	Group-III	Group-IV	Group-V	*p* Value
IL-6	0 (0–1)	1 (1–2)	2 (1–3)	2 (1–3)	1 (1–2)	0.001
IL-8	0.5 (0–1)	1.5 (1–2)	2 (1–3)	1.5 (1–2)	2 (1–3)	0.005
IL-10	0 (0–1)	1 (1–2)	1.5 (1–3)	2 (1–3)	1.5 (1–2)	<0.001
TNF-α	0 (0–1)	1 (1–2)	2 (1–3)	2 (1–3)	1.5 (1–2)	0.001

**Table 4:** *p* values of the IHC staining regarding cytokine expressions assessed by the Mann–Whitney U analysis based on paired group comparisons.

Cytokine	Group I/Group II (NC/VC)	Group I/Group III (NC/HT480)	Group I/Group IV (NC/M1)	Group I/Group V (NC/YF1B-b)
IL-6	0.002	0.001	0.001	0.003
IL-8	0.010	0.004	0.006	0.004
IL-10	0.001	0.001	<0.001	0.001
TNF-α	0.005	0.001	0.002	0.003

**Table 5:** Effects of the group and the measurement time on the ELISA results regarding IL-6, IL-8, IL-1,0 and TNF-α cytokine levels in the sera.

		Group I (NC)	Group II (VC)	Group III (HT480)	Group IV (M1)	Group V (YF1B-b)		*p* Values in Repeated ANOVA Statistics		
Cytokine	Time Points	Mean ± SE	Mean ± SE	Mean ± SE	Mean ± SE	Mean ± SE	*p*-Values in One-Way ANOVA	Group	Measurement Time	Group × Measurement Time
IL-6	1 (3 h)	10.30 ± 0.63	10.88 ± 1.52	17.24 ± 2.04	12.83 ± 1.58	15.41 ± 1.65	0.014	0.004	0.025	0.233
	2 (6 h)	10.94 ± 0.77	12.27 ± 0.87	25.69 ± 6.44	18.44 ± 2.29	15.25 ± 1.25	0.017			
	Significance	0.108	0.420	0.162	0.071	0.955				
IL-8	1 (3 h)	11.83 ± 2.22	23.22 ± 13.24	72.24 ± 3.81	69.14 ± 4.71	74.67 ± 4.39	<0.001	<0.001	0.297	0.085
	2 (6 h)	11.76 ± 2.37	26.85 ± 3.26	73.90 ± 3.72	66.59 ± 2.54	61.72 ± 3.03	<0.001			
	Significance	0.808	0.205	0.637	0.686	0.079				
IL-10	1 (3 h)	2.69 ± 0.48	17.18 ± 3.36	41.43 ± 5.95	38.77 ± 8.66	41.27 ± 6.42	<0.001	<0.001	0.411	0.970
	2 (6 h)	2.49 ± 0.44	17.81 ± 3.95	45.71 ± 8.64	46.45 ± 5.43	45.01 ± 8.44	<0.001			
	Significance	0.100	0.739	0.656	0.487	0.787				
TNF-α	1 (3 h)	6.22 ± 1.09	27.61 ± 5.32	86.81 ± 10.31	109.63 ± 18.17	122.77 ± 36.67	<0.001	<0.001	0.004	0.033
	2 (6 h)	6.45 ± 1.02	31.91 ± 5.49	178.55 ± 29.66	143.08 ± 14.72	141.22 ± 10.98	<0.001			
	Significance	0.558	0.193	0.011	0.010	0.650				

## Data Availability

Not applicable.
